# Maternal gene polymorphisms of folate metabolism as genetic risk factor for Down syndrome in North Indian population

**DOI:** 10.1186/1755-8166-7-S1-P120

**Published:** 2014-01-21

**Authors:** Sushil Kumar Jaiswal, Ashok Kumar, Vineeta Gupta, Anjali Rani, Amit Kumar Rai

**Affiliations:** 1 Centre for Genetic Disorders, Institute of Medical Sciences, Banaras Hindu University, Varanasi-221005, India; 2 Department of Pediatric, Institute of Medical Sciences, Banaras Hindu University, Varanasi-221005, India; 3 Department of Gynaecology, Institute of Medical Sciences, Banaras Hindu University, Varanasi-221005, India

## 

Down syndrome (DS), a chromosomal disorder has higher prevalence in population occurring 1 in 700 live births. Recent reports have shown that almost 92% of the DS children are born from young mothers, suggesting that along with advanced maternal age some other risk factors are involved for predisposition of mother to Down child. Polymorphism in genes involved in folate metabolism as well as insufficient folic acid intake could result in genomic instability, DNA hypomethylation and non-disjunction even resulting in trisomy 21. In present study we compared the frequency of Thymidylate Synthase (*TYMS*) 28 bp repeat polymorphism in 5’UTR region, Cystathionine-Beta-Synthase (*CBS*) 844ins68bp polymorphism and Solute Carrier (*SLC*) 19A1 G80A single nucleotide polymorphisms in 80 triads (mother, father and child) and 77 matched control mothers in order to observe whether these variants act as risk factors for DS. A significant association was observed for TYMS 5’UTR 28 bp repeat with odds ratio 2.9 (95% CI 1.2-7.1, p=0.027). An association which is very close to be significant was observed for SLC19A1 G80A with odds 2.01(95% CI 1.04-4.24, p=0.055). Heterozygosity for 68 bp insertion at 844 in CBS showed significant association with odds ratio 10.5 (95% CI 1.29-85.1, p=0.019). Transmission disequilibrium test (TDT) for 28 bp repeat polymorphism in TYMS gene presented more than four times greater preferential transmission of maternal two repeats allele whereas paternal three repeats allele had about 1.5 times higher rate of transmission. TDT for SLC19 A1 G80A SNPs revealed preferential transmission of maternal A allele more than two times greater as compared to G allele whereas paternal alleles transmission didn’t show much difference. The result shows that above three polymorphism are significantly associated as a risk factor for predisposition of mother to DS children in the North Indian population.

